# Acoustic Structure and Contextual Use of Calls by Captive Male and Female Cheetahs (*Acinonyx jubatus*)

**DOI:** 10.1371/journal.pone.0158546

**Published:** 2016-06-30

**Authors:** Darya S. Smirnova, Ilya A. Volodin, Tatyana S. Demina, Elena V. Volodina

**Affiliations:** 1 Department of Animal Science, Russian State Agrarian University—Moscow Timiryazev Agricultural Academy, Moscow, Russia; 2 Department of Vertebrate Zoology, Faculty of Biology, Lomonosov Moscow State University, Moscow, Russia; 3 Scientific Research Department, Moscow Zoo, Moscow, Russia; University of Pavia, ITALY

## Abstract

The vocal repertoire of captive cheetahs (*Acinonyx jubatus*) and the specific role of meow vocalizations in communication of this species attract research interest about two dozen years. Here, we expand this research focus for the contextual use of call types, sex differences and individual differences at short and long terms. During 457 trials of acoustic recordings, we collected calls (n = 8120) and data on their contextual use for 13 adult cheetahs (6 males and 7 females) in four Russian zoos. The cheetah vocal repertoire comprised 7 call types produced in 8 behavioural contexts. Context-specific call types (chirr, growl, howl and hiss) were related to courting behaviour (chirr) or to aggressive behaviour (growl, howl and hiss). Other call types (chirp, purr and meow) were not context-specific. The values of acoustic variables differed between call types. The meow was the most often call type. Discriminant function analysis revealed a high potential of meows to encode individual identity and sex at short terms, however, the vocal individuality was unstable over years. We discuss the contextual use and acoustic variables of call types, the ratios of individual and sex differences in calls and the pathways of vocal ontogeny in the cheetah with relevant data on vocalization of other animals.

## Introduction

Cheetahs (*Acinonyx jubatus*) are among animals that are most attractive for people due to their nice appearance and interesting communicative behaviour with conspecifics and humans [1,2]. The cheetahs were intensely studied in relation to their wildlife ecology [3–7], conservation [8–10], diseases [11], morphology [12–14] and genetics [15–17]. At the same time, the acoustic communication is rather poorly investigated for the cheetah.

**Fig 1:**
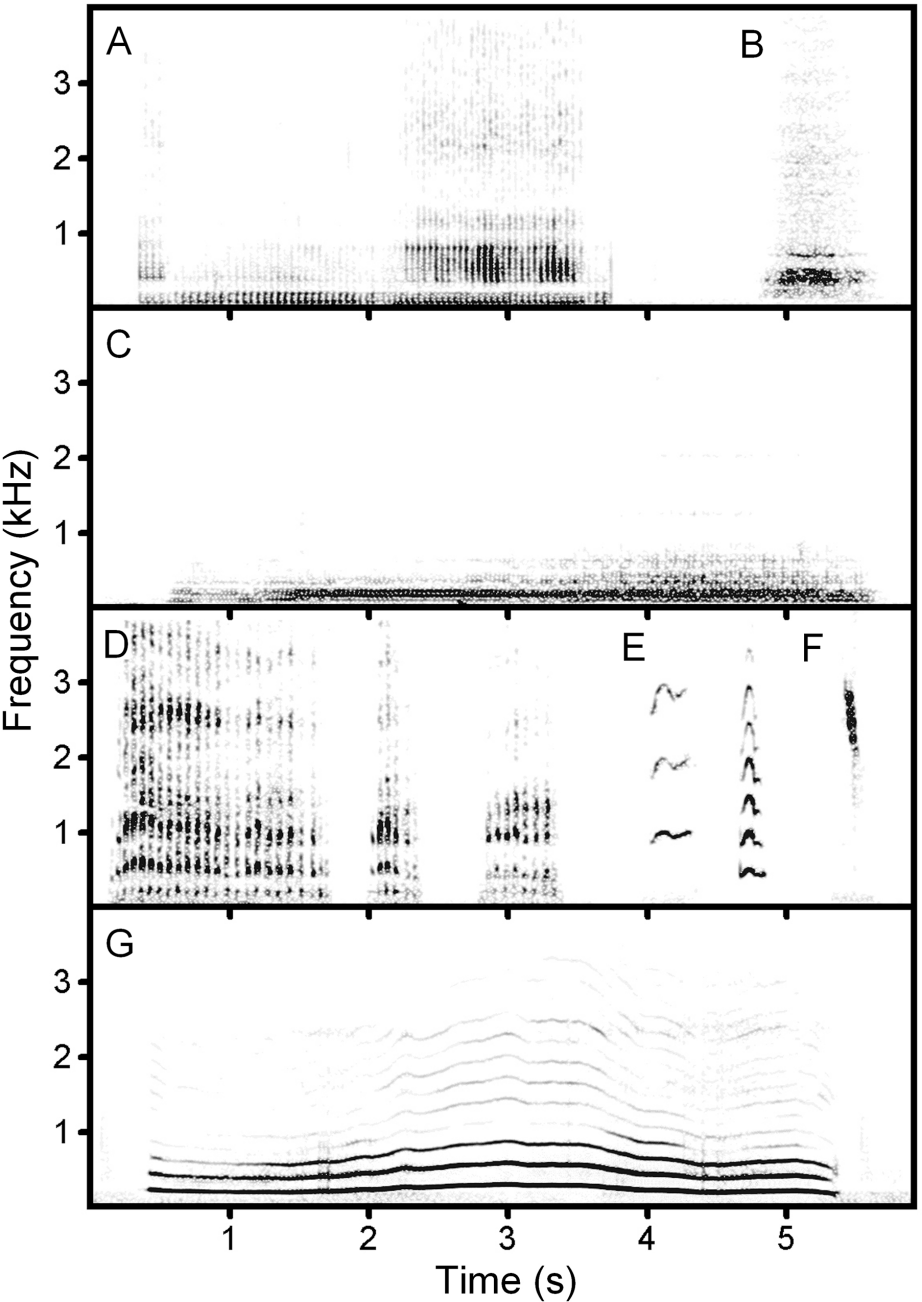
Spectrogram illustrating seven call types produced by adult (male and female) cheetahs (*Acinonyx jubatus*) in captivity. A–purr, B–hiss, C–growl, D–chirr, E–meow, F–chirp, G–howl (S1 Audio). The spectrogram was created at 11025 Hz sampling frequency, Fast Fourier Transform (FFT) 1024, Hamming window, frame 50%, overlap 93.75%.

Previously, based on the acoustic structure, call types of the vocal repertoire have been described for cheetah cubs [18] and for cheetah adults [19]. The vocal repertoire of adult cheetahs comprises eight call types: purr, hiss, growl, chirr, meow, chirp, howl and gurgle [19] (Fig 1 and S1 Audio). In cheetah cubs younger three months, the vocal repertoire comprised of the same seven call types as in adults, for the exclusion of the gurgle [18]. For assessing the functional role of different call types in the cheetah, a hypothetical scheme relating the acoustic structure with emotional states of confidence/diffidence and aggressiveness/non-aggressiveness, has been proposed [19]. However, this scheme has not yet been confirmed with quantitative material on the contextual use of calls.

Whereas the study by Volodina [19] was related to the entire vocal repertoire of adult cheetahs, all other studies of cheetah vocalizations were related to particular call types within the cheetah vocal repertoire. On example of cheetah purr and chirr vocalizations, the mechanism of vocal vibration, representing an uninterrupted emission of pulsed vocalization for the duration of both inspiration and expiration phases of breathing, has been investigated [20]. Purring is caused by rapid twitching of the vocalis muscle, whereas the chirr vocalization is caused with interaction between the purr and the tonal vocalization produced by the normal vibration of the vocal folds [20,21]. The acoustics of cheetah purr vocalizations, produced continuously during the inspiration and expiration phases, were also examined in a few studies [19,22–24]. The acoustics of agonistic vocalizations of the cheetah have been considered by Eklund with coauthors [25]. Frustrative meows of captive adult male and female cheetahs and of cheetah cubs, were preliminary described in [26]. The studies focused on searching the vocal indicators of reproductive state in the cheetah [27,28] revealed that males produce a specific “courting” series of chirrs interspersed with chirps when exposed to urine samples taken from receptive females.

While the bark represents the most characteristic vocalization of canids [29–36], the meow represents the most characteristic vocalization of felids, either wild [37–39] or domesticated [38–43]. Consistently, the meow represents a prominent vocalization in their vocal repertoire of the cheetah [18,19,26]. Cheetah meows occur in different contexts but primarily in the contexts related to frustration and discomfort (solicitation, anticipation and separation) [26,44,45]. Previously, vocal individuality of meow calls was shown in the isolation context for domestic kittens (*Felis catus*) [42] and for four adult male cheetahs [44]. At the same time, sex-specific variation of vocalizations was not yet studied for any felid species, although some vocal sex dimorphism is expected given the sexual dimorphism in body size. For example, approximately 20% difference in body mass exists between male and female domestic cats [46], 15% between male and female captive cheetahs [8] and 22% between male and female wild cheetahs [14]. Therefore, the vocal apparatus and the sound-producing structures are also expected to be larger for the larger sex [47,48] and their acoustics (the fundamental and formant frequencies) are expected to be lower in the larger sex [49,50]. However, very close or indistinguishable acoustics between vocalizations of the larger and smaller sex were reported for some subspecies of red deer (*Cervus elaphus hispanicus* [51] and *C*. *e*. *sibiricus* [52]).

The contextual use of different call types represents the powerful tool for examining the functions of these calls [53]. For the cheetah, the occurrence of different call types across behavioural contexts in captivity was examined only preliminary [45]. The focus of this study is to integrate the structural and contextual analysis of cheetah vocalizations for more precise assessment of the functional role of different call types in this species. The purposes of this study were: 1) to estimate quantitatively the contextual use of different call types by adult captive cheetahs across behavioural contexts, 2) to estimate quantitatively the identity and sex-related differences in meows at short terms and 3) to estimate the stability of vocal individual traits in cheetah meows over years.

## Material and Methods

### Ethics statement

Three authors (IAV, EVV and TSD) are zoo staff members, so no special permission was required for them and for their Master’s student (DSS) to work with animals in Moscow Zoo and Volokolamsk Zoo Breeding Station. For access to the cheetahs kept in Yaroslavl and Novosibirsk zoos, the specific permissions were obtained from administration of these zoos by the request from the President Director of Moscow Zoo V.V. Spitsin for the period of data collection. Vocalizations were recorded from inside and outside the animal enclosures during zoo working hours under supervision of zoo staff. Call collector (DSS) did not manipulate the animals for the purpose of this study. Disturbance of animals was kept to a minimum. No animal has suffered somehow due to the data collection. The research protocol # 2011–36 has been approved by the Committee of Bio-ethics of Lomonosov Moscow State University. We adhered to the ‘Guidelines for the treatment of animals in behavioural research and teaching’ (Anim. Behav., 2006, 71, 245–253) and to the laws on animal welfare for scientific research of the Russian Federation, where the study was conducted.

### Study subjects

Spontaneously produced calls of 13 (6 male and 7 female) adult (older 2 years) captive-born cheetahs of the African subspecies (*A*. *j*. *jubatus*) were recorded in 2012–2014 in zoos of Russia.

In particular, six animals (Male 1 “Seva”, Male 2 “Adam”, Male 3 “Kay”, Female 7 “Eva”, Female 8 “Sindi”, Female 9 “Kimi”) were recorded at Volokolamsk Zoo Breeding Station of Moscow Zoo (Moscow region, Volokolamsk district) in May-August 2012. Five of these animals (Males 1, 2 and Females 7, 8, 9) were then repeatedly recorded in June-July 2014 and their calls served for analysis of the stability of the individual acoustic characteristics with time.

Two animals (Male 4 “Adday”, Female 10 “Nayla”) were recorded in Yaroslavl Zoo (Jaroslavl city) in June 2013. Two animals (Male 5 “Kidjan”, Female 11 “Annay”) were recorded in Novosibirsk Zoo (Novosibirsk city) in July 2013. Three animals (Male 6 “Frank”, Female 12 “Zygota”, Female 13 "Winda") were recorded in Moscow Zoo (Moscow city) in October-November 2014.

### Animal housing

Animals were kept in outdoor enclosures (sizes varied from 240 to 500 m^2^ depending on the zoo) containing the indoor enclosures (warm houses subdivided inside into four departments 4x4 m, individual for each animal). During the day, animals were released into the outdoor enclosure (together or singly, depending on the zoo), whereas during the night they stayed inside their individual indoor enclosures. The walls of the indoor enclosures were made of wire-mesh, so the animals could see the conspecifics in the indoor enclosures. The feeding occurred twice a day in the individual indoor enclosures, the first one in the morning before the releasing to the outdoor enclosures and the second one in the evening, before the locking the exit for the night.

### Call collection

Calls were recorded indoor and outdoor. The call collector (DSS) was in the same indoor or outdoor enclosure as animals, but was separated from them with wire-mesh and not entered into contact with animals. Calls were collected by the focal animal sampling method [54]. For sound recordings (sampling rate 48 kHz, 16 bit resolution) we used a Zoom-H4n professional digital recorder (Zoom Corporation, Tokyo, Japan) with built-in microphones. All acoustic recordings were conducted during daytime in periods of maximal activity of the animals during routine procedures (feeding, releasing from indoor to outdoor enclosures, communication of animals with their keepers and with other cheetahs through the wire-mesh).

We established eight mutually exclusive behavioural contexts in which calls were produced: 1) "Offensive" context (attack or threat aggression toward a human, researcher or keeper, or toward a conspecific); 2) "Defensive" context (defensive aggression toward a human or conspecific); 3) "Conspecific-Contact" context (friendly close-range communication between conspecifics); 4) "Call-Over" context (distant communication with a conspecific, commonly without visual contact); 5) "Human-Contact" context (friendly close-range communication with a human, researcher or keeper); 6) "Release-Soliciting" context (keeper-directed soliciting to release the animal outdoor or back; 7) "Food-Anticipating" context (feeding anticipation and arousal when seeing the food and feeding the neighbor animals; 8) "Courting" context (male courting female as a component of sexual behaviour). Calls were not used as markers for identifying the contexts. During the recording, the researcher labeled the context by voice. Individuals could be distinguished by their coloration pattern. The distance from microphones to the animals was 0.5–10 m. Each recording trial (457 trials in total) lasted 1–10 minutes, contained calls from a single individual cheetah and was stored as a wav-file. The number of trials per cheetah individual was from 6 to 86, on average 35.2 ± 23.7 trials per individual.

### Call samples

We tried to collect material maximally balanced by individuals and contexts. However, as vocal activity of different individuals was unequal, call samples were unevenly distributed by different animals. Most indoor recordings contained echo; this did not interfere determining call types but limited the number of calls potentially applicable for analysis of the acoustic structure. We prepared four different call samples: 1) for analysis of the occurrence of call types in different behavioural contexts, 2) for describing the acoustics of call types, 3) for estimating the effects of sex and individual identity on the acoustics of meows and 4) for estimating the stability of the acoustic individuality in meows with time.

For analysis of the occurrence of call types in different behavioural contexts, we analysed a total of 8120 cheetah calls for their call type using the Avisoft SASLab Pro software (Avisoft Bioacoustics, Berlin, Germany). Based on frequency and temporal acoustic structure, we subdivided calls into seven types: purr, hiss, growl, chirr, meow, chirp and howl (Fig 1), following the early classification [19]. In cases when calls had a transitional acoustic structure [19], e.g. from meow to purr or from howl to growl, each call part was treated as a separate call. For each call, we determined the behavioural context (among the eight contexts) in which it was produced. Then we examined the contexts in which the 8120 calls were used, following the criteria previously adopted by Salmi et al. [53]. We classified a call type as context-specific if it was given in the same behavioral context more than 65% of cases. Because multiple call types can be used in the same context, we then examined whether this was the primary call type for this context, classifying it as signal-specific if it accounted for more than 65% of all calls given during that context [53].

For describing the acoustics of call types, occurring in cheetahs in captivity, we selected from the total massive of recordings from 6 to 60 calls per each of the 7 call types, 183 calls in total. For each call type, calls were taken from 3 to 12 animals. We took calls of best quality, not superimposed with other calls and background noise.

For estimating the effects of sex and individuality on the acoustic variables of meows, we selected 10–15 calls per individual from 12 individuals, for the exclusion of Female 13, which did not produce meows, 151 meows in total. To decrease the effect of pseudoreplication, we selected calls from different recording trials and within recording trials calls from different parts of the trial, avoiding taking calls following one after other.

For estimating the stability of vocal individuality in meows with time (over 2 years), we selected 10–15 meows per individual from 5 individuals (Males 1, 3 and Females 7, 8, 9) recorded in 2012 (69 meows) and in 2014 (70 meows), 139 meows in total.

### Call analysis

Only calls with high call-to-noise ratio, non-overlapped with background noise or calls of other individuals, non-disrupted by wind, and clearly identified as belonging to focal individuals were included in the analysis. For call analysis, we used Avisoft, with 48 kHz sampling frequency, the Hamming window, FFT length 1024 points, frame 50% and overlap 93.75%. These settings allowed frequency resolution 46 Hz and time resolution 1.3 ms. All measurements were made manually and have been exported to Microsoft Excel (Microsoft Corp., Redmond, WA, USA).

**Fig 2:**
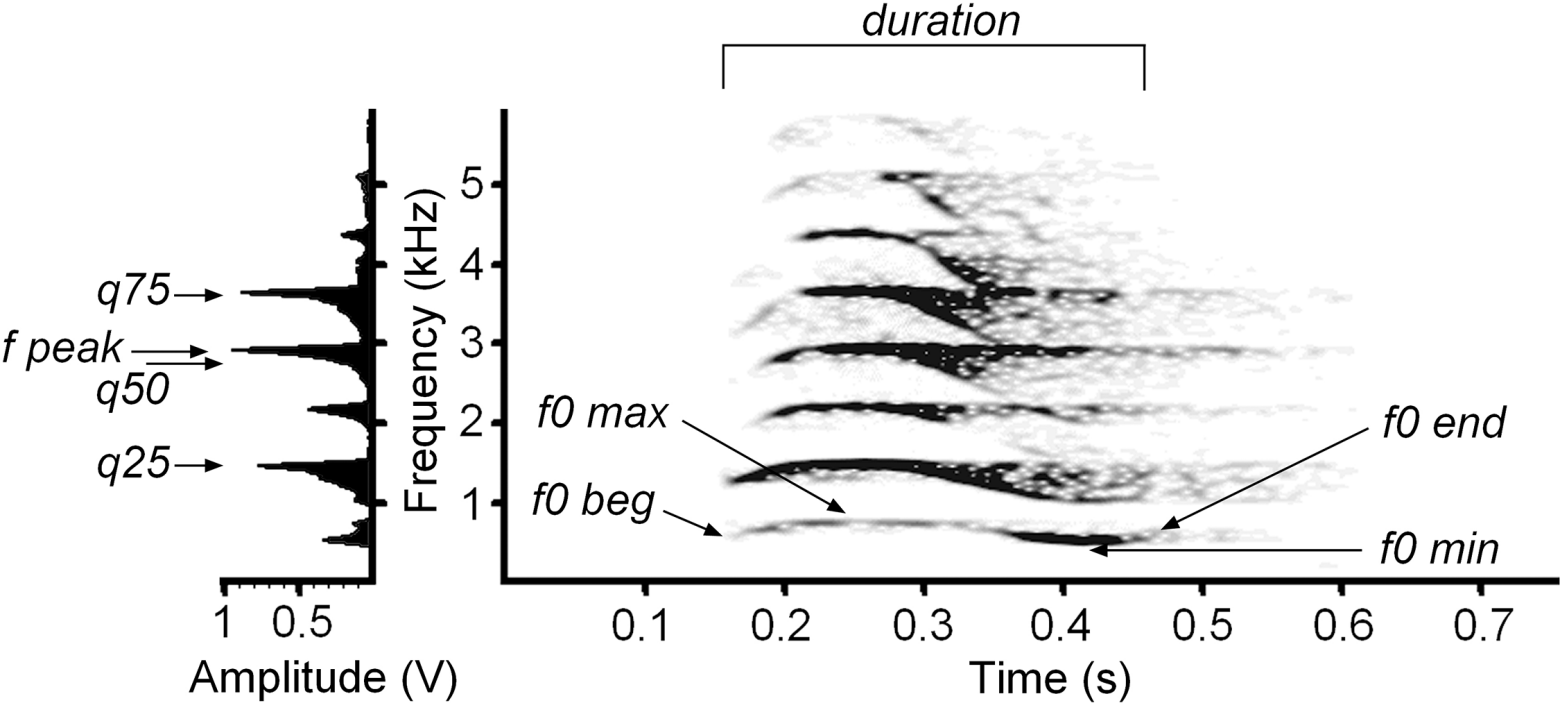
Measured variables for cheetah meows. Spectrogram (right) and mean power spectrum of the entire call (left). Designations: duration–call duration; f0 beg–the fundamental frequency at the onset of a call; f0 end–the fundamental frequency at the end of a call; f0 max–the maximum fundamental frequency; f0 min–the minimum fundamental frequency; f peak–the frequency of maximum amplitude within a call; q25, q50 q75 –the lower, the medium and the upper quartiles, covering respectively 25%, 50% and 75% energy of a call spectrum. The spectrogram was created at 11025 Hz sampling frequency, Fast Fourier Transform (FFT) 512, Hamming window, frame 50%, overlap 96.87%.

For calls of all types, we measured the duration from the screen with the standard marker cursor in the spectrogram window (Fig 2), for the exclusion of purr, whose duration could last many minutes. In addition, for all calls of all types, we measured the maximum amplitude frequency (f peak) and three quartiles (q25, q50 and q75), covering respectively 25, 50 and 75% of call energy (hereafter the lower, medium and upper quartiles) from the mean power spectrum of each call. For purr, produced continuously at both respiratory phases, the acoustic variables were measured separately for the inspiration and the expiration call phases. In calls with rhythmic pulsation (chirr, growl and purr call types) we also measured with the standard marker cursor the pulse rate. For each purr vocalization, three subsequent phases of expiration-inspiration were included in analyses for calculating power variables and pulse rate. In growl, meow, chirp and howl calls we additionally measured, with the reticule cursor, the initial (f0 beg), end (f0 end), maximum (f0 max) and minimum (f0 min) fundamental frequencies of each call (Fig 2).

The acoustic measurements for describing the acoustics of call types are presented in S1 Table. The acoustic measurements for estimating the effects of sex and individuality on the acoustic variables of meows and for estimating the stability of vocal individuality in meows with time are presented in S2 Table.

### Statistical analyses

Statistical analyses were made with STATISTICA, v. 6.0 (StatSoft, Tulsa, OK, USA) and R v.3.0.1 [55]; all means are given as mean ± SD. Significance levels were set at 0.05, and two-tailed probability values are reported. Only 20 of 224 distributions of measured parameter values did depart from normality (Kolmogorov-Smirnov test, *p* > 0.05) what allowed us to apply parametric tests.

We used a two-way ANOVA with Tukey HSD test to compare the acoustics among call types, with call type as fixed factor and individual as random factor. We used a two-way ANOVA with Tukey HSD test to compare the acoustics between purr inspiration and expiration phases, with phase as fixed factor and individual as random factor. We used a nested design of ANOVA with an individual nested within sex to estimate effects of factors “individuality” and “sex”, on the acoustic variables of meows, with sex as fixed factor and individual as random factor.

We used standard procedure of discriminant function analysis (DFA) to calculate the probability of the assignment of meows to the correct individual. Nine variables, used for the DFA, showed very low Pearson correlation values to each other. Among the total of 36 pairwise correlations, the R^2^ values were lower 0.2 for 23 comparisons; between 0.2 and 0.4 for 3 comparisons; between 0.4 and 0.6 for 4 comparisons; between 0.6 and 0.8 for 5 comparisons, and only for 1 comparison (f0 end with f0 min) the R^2^ value was 0.91. Then we investigated the stability of acoustic individuality of meows between years for cheetahs that provided calls in two years. We classified meows from 2014 with DFA functions derived from 2012, considering the value of the correct cross-validation as a measure of the retention of individuality over time [56–58]. With a 2x2 Yates' chi-squared test, we compared the values of correct assignment of meows to the correct caller between years.

We used Wilks’ Lambda values to estimate how strongly acoustic variables of calls contribute to discrimination of individuals. To validate our DFA results, we calculated the random values of correct assignment of calls to individual by applying randomization procedure with macros, created in R. The random values were averaged from DFAs performed on 1000 randomized permutations on the data sets as described by [59]. Using a distribution obtained by the permutations, we noted whether the observed value exceeded 95%, 99% or 99.9% of the values within the distribution [59]. If the observed value exceeded 95%, 99% or 99.9% of values within this distribution, we established that the observed value did differ significantly from the random one with a probability *p* < 0.05, *p* < 0.01 or *p* < 0.001 respectively [57–60].

## Results

### Acoustic structure of cheetah calls

From the total sample of cheetah calls, 7228 calls could be classified to distinctive call types, whereas 446 calls (5.81%, N = 7674 calls) were transitional from one call type to another (thus including two different call types, one after another without time space between them). In these cases each call part of the transitional call was treated as a separate call, what resulted in the total sample of 8120 calls analysed for call type. Most often transitional calls occurred between purr and meow (267 calls), growl and howl (86 calls) and growl and meow (60 calls).

Based on ANOVA results, we found that all acoustic variables were significantly related to call type (Table 1). The duration did not differ between the growl and howl, being significantly higher than in all other call types for the exclusion of purr, the continuous vocalization whose duration could not be measured. The duration did not differ significantly between the chirr, meow, chirp and hiss call types (Table 1 and Fig 1).

**Table 1:** Values (mean±SD) of acoustic variables for the cheetah call types.

Acoustic	Call type							ANOVA
variable	Chirr (N = 11)	Purr (N = 11)	Growl (N = 33)	Meow (N = 60)	Chirp (N = 6)	Howl (N = 19)	Hiss (N = 43)	
Duration (s)	0.74±0.73 ^a^		2.34±1.31 ^b^	0.32±0.14 ^a^	0.11±0.04 ^a^	2.11±1.28 ^b^	0.62±0.20 ^a^	*F*_5,154_ = 38.01; *р*<0.001
Pulse rate (Hz)	16.59±1.03 ^a^	22.68±2.67 ^b^	37.40±4.28 ^c^	-	-	-	-	*F*_2,44_ = 98.46; *р*<0.001
f0 beg (kHz)	-	-	0.16±0.09 ^a^	0.81±0.31 ^b^	1.81±0.65 ^c^	0.26±0.10 ^a^	-	*F*_3,102_ = 69.85; *р*<0.001
f0 end (kHz)	-	-	0.15±0.03 ^a^	0.69±0.24 ^b^	0.89±0.39 ^c^	0.25±0.09 ^a^	-	*F*_3,102_ = 67.76; *р*<0.001
f0 max (kHz)	-	-	0.19±0.08 ^a^	0.94±0.35 ^b^	1.81±0.65 ^c^	0.31±0.14 ^a^	-	*F*_3,102_ = 74.29; *р*<0.001
f0 min (kHz)	-	-	0.14±0.04 ^a^	0.68±0.23 ^b^	0.89±0.39 ^c^	0.21±0.06 ^a^	-	*F*_3,102_ = 77.18; *р*<0.001
f peak (kHz)	0.59±0.33 ^a^	0.16±0.14 ^b^	0.21±0.11 ^b^	1.07±0.40 ^c^	1.76±0.60 ^d^	0.27±0.10 ^b^	0.32±0.11 ^b^	*F*_6,157_ = 77.58; *р*<0.001
q25 (kHz)	0.68±0.26 ^a^	0.14±0.11 ^b^	0.19±0.08 ^b^	1.01±0.32 ^c^	1.63±0.61 ^d^	0.24±0.10 ^b,e^	0.42±0.25 ^e^	*F*_6,164_ = 80.26; *р*<0.001
q50 (kHz)	1.19±0.36 ^a,d^	0.32±0.14 ^b^	0.34±0.16 ^b^	1.52±0.42 ^a,c^	1.88±0.61 ^c^	0.44±0.31 ^b^	1.01±0.72 ^d^	*F*_6,164_ = 40.18; *р*<0.001
q75 (kHz)	2.03±0.44 ^a^	0.88±0.50 ^b^	0.88±0.77 ^b^	2.14±0.73 ^a^	2.44±0.42 ^a^	0.99±0.91 ^b^	2.30±1.01 ^a^	*F*_6,164_ = 24.88; *р*<0.001

Results for comparison of acoustics between call types (two-way ANOVA with Tukey HSD test with call type as fixed factor and individual identity as random factor) are given with letters; means sharing the same letter are not significantly different. N = total number of calls of each type.

The pulse rate was minimal in the chirr, intermediate in the purr and maximal in the growl call type; all differences were found significant. All fundamental frequency variables did not differ between the growl and howl call types, being significantly lower than for the meow and chirp call types and for the meow significantly lower than for the chirp (Table 1, Fig 1).

The values of the peak frequency and of the three power quartiles were the highest for the chirp, lower for the meow and more prominently lower for the chirr. For the purr, growl, howl and hiss, the values of the peak frequency and of the three power quartiles were the lowest ones and differed significantly from the three other call types (for the exclusion of q50 and q75 for the hiss) (Table 1).

For the purr, we also compared the values of acoustic variables between the inspiration and expiration phases (Table 2). The values of the pulse rate, the peak frequency, and the lower and medium quartiles were significantly higher for the inspiration phase than for the expiration phase, whereas the values of the upper quartile did not differ between the inspiration and expiration phases of the purr call type (Table 2).

**Table 2:** Values (mean±SD) of acoustic variables for the cheetah purr inspiration and expiration phases.

Acoustic variable	Inspiration (N = 11)	Expiration (N = 11)	ANOVA
Pulse rate (Hz)	24.57±2.05	20.80±1.74	*F*_1,18_ = 64.65; *р*<0.001
f peak (kHz)	0.25±0.15	0.08±0.03	*F*_1,14_ = 18.129; *р*<0.001
q25 (kHz)	0.21±0.11	0.08±0.03	*F*_1,18_ = 22.58; *р*<0.001
q50 (kHz)	0.43±0.05	0.21±0.11	*F*_1,18_ = 40.68; *р*<0.001
q75 (kHz)	0.91±0.22	0.84±0.69	*F*_1,18_ = 0.09; *р =* 0.77

Note: Three subsequent phases of expiration-inspiration measured for each poor vocalization (N = 11) served for calculating the acoustic variables.

### Context and signal-specific call types

Four of 7 call types were context-specific (i.e., given mostly in one specific context), including growl, howl and hiss (during Offensive context) and chirr (during Courting context) (Table 3). All context-specific calls were given in a single context more than in 80% cases, except for howl, which were given in a context-specific way in 72.2% cases. The three remaining call types (meow, chirp and purr) were not found context-specific. The meow was the single call type occurring in all behavioural contexts (Table 3).

**Table 3:** Call context-specificity: the percent of calls given in each of context, context-specific calls (i.e., for which > 65%) are indicated in bold.

Call	Context								
type	N_VOC_	Offensive	Defensive	Conspecific-Contact	Call-Over	Human-Contact	Release-Soliciting	Food-Anticipating	Courting
Chirr	978	0	0.2	0	0	2.2	0	0.1	**97.4**
Purr	1045	0	0	1.1	0.5	48.0	2.8	47.7	0
Growl	1485	**81.1**	9.6	0	0	0	1.9	7.3	0.2
Meow	3867	0.6	0.1	1.3	5.7	1.9	37.1	45.6	7.8
Chirp	73	1.4	2.7	0	26.0	1.4	52.1	8.2	8.2
Howl	212	**72.2**	6.6	0	0	0	8.0	13.2	0
Hiss	460	**87.8**	8.9	0	0	0	0	3.3	0
N_BC_	8120	1786	204	60	243	598	1548	2418	1263

N_VOC_ = total number of calls of each type recorded in all contexts; N_BC_ = total number of calls given in each behavioral context.

For each of the 8 behavioural contexts we found the signal-specific call types (i.e., the most common call type used in a specific context) (Table 4). The chirr was given primarily during the Courting context; the purr was given primarily during the Human-Contact context. The growl was given primarily during two aggressive contexts: the Offensive and the Defensive. The meow usage was specific during four contexts: the Conspecific-Contact, the Call-Over, the Release-Soliciting and the Food-Anticipation. The remaining three call types (chirp, howl and hiss) were not signal-specific (Table 4).

**Table 4:** Call signal-specificity: the percent of 7 call types given in each context, signal-specific calls (i.e., those for which > 65% are given in a single context) are indicated in bold.

Context		Call type						
	N_BC_	Chirr	Purr	Growl	Meow	Chirp	Howl	Hiss
Offensive	1786	0	0	**67.4**	1.3	0.1	8.6	22.6
Defensive	204	1.0	0	**69.6**	1.5	1.0	6.9	20.1
Conspecific-Contact	60	0	18.3	0	**81.7**	0	0	0
Call-Over	243	0	2.1	0	**90.1**	7.8	0	0
Human-Contact	598	3.7	**83.9**	0	12.2	0.2	0	0
Release-Soliciting	1548	0	1.9	1.8	**92.8**	2.5	1.1	0
Food-Anticipating	2418	0.1	20.6	4.5	**72.9**	0.2	1.2	0.6
Courting	1263	**75.5**	0	0.2	23.8	0.5	0	0
N_VOC_	8120	978	1045	1485	3867	73	212	460

N_VOC_ = total number of calls of each type recorded in all contexts; N_BC_ = total number of calls given in each behavioral context.

### Effects of identity and sex on meow acoustics

Two-way ANOVA revealed effects of individual identity on all variables of meows, whereas effects of sex were found only on variables of fundamental frequency (Table 5). Values of all fundamental frequency variables were substantially and significantly lower in males than in females (for instance, the f0 max was 0.85±0.40 kHz in males and 1.07±0.25 kHz in females, Table 5).

**Table 5:** Values (meanSD) of the cheetah meow variables and results of nested ANOVA for individual and sex differences.

Acoustic	ANOVA		Variable values for each sex	
variable	Individual differences	Sex differences	Male calls (N = 71)	Female calls (N = 80)
Duration (s)	*F*_10,139_ = 5.43; *р*<0.001	*F*_1,139_ = 0.54; *р* = 0.46	0.31±0.17	0.34±0.14
f0 beg (kHz)	*F*_10,139_ = 4.79; *р*<0.001	*F*_1,139_ = 40.19; *р*<0.001	0.68±0.29	0.92±0.22
f0 end (kHz)	*F*_10,139_ = 12.01; *р*<0.001	*F*_1,139_ = 33.54; *р*<0.001	0.62±0.25	0.77±0.16
f0 max (kHz)	*F*_10,139_ = 13.18; *р*<0.001	*F*_1,139_ = 29.30; *р*<0.001	0.85±0.40	1.07±0.25
f0 min (kHz)	*F*_10,139_ = 9.90; *р*<0.001	*F*_1,139_ = 40.83; *р*<0.001	0.59±0.22	0.76±0.16
f peak (kHz)	*F*_10,139_ = 11.52; *р*<0.001	*F*_1,139_ = 0.28; *р* = 0.60	1.22±0.74	1.11±0.35
q25 (kHz)	*F*_10,139_ = 20.27; *р*<0.001	*F*_1,139_ = 0.01; *р* = 0.91	1.04±0.51	1.00±0.20
q50 (kHz)	*F*_10,139_ = 9.32; *р*<0.001	*F*_1,139_ = 1.62; *р* = 0.21	1.58±0.63	1.43±0.38
q75 (kHz)	*F*_10,139_ = 4.35; *р*<0.001	*F*_1,139_ = 0.0; *р* = 0.98	2.38±0.79	2.31±0.64

Note: Individual nested within sex (with sex as fixed factor, and individual as random factor); N = 12 cheetahs (6 males and 6 females).

**Fig 3:**
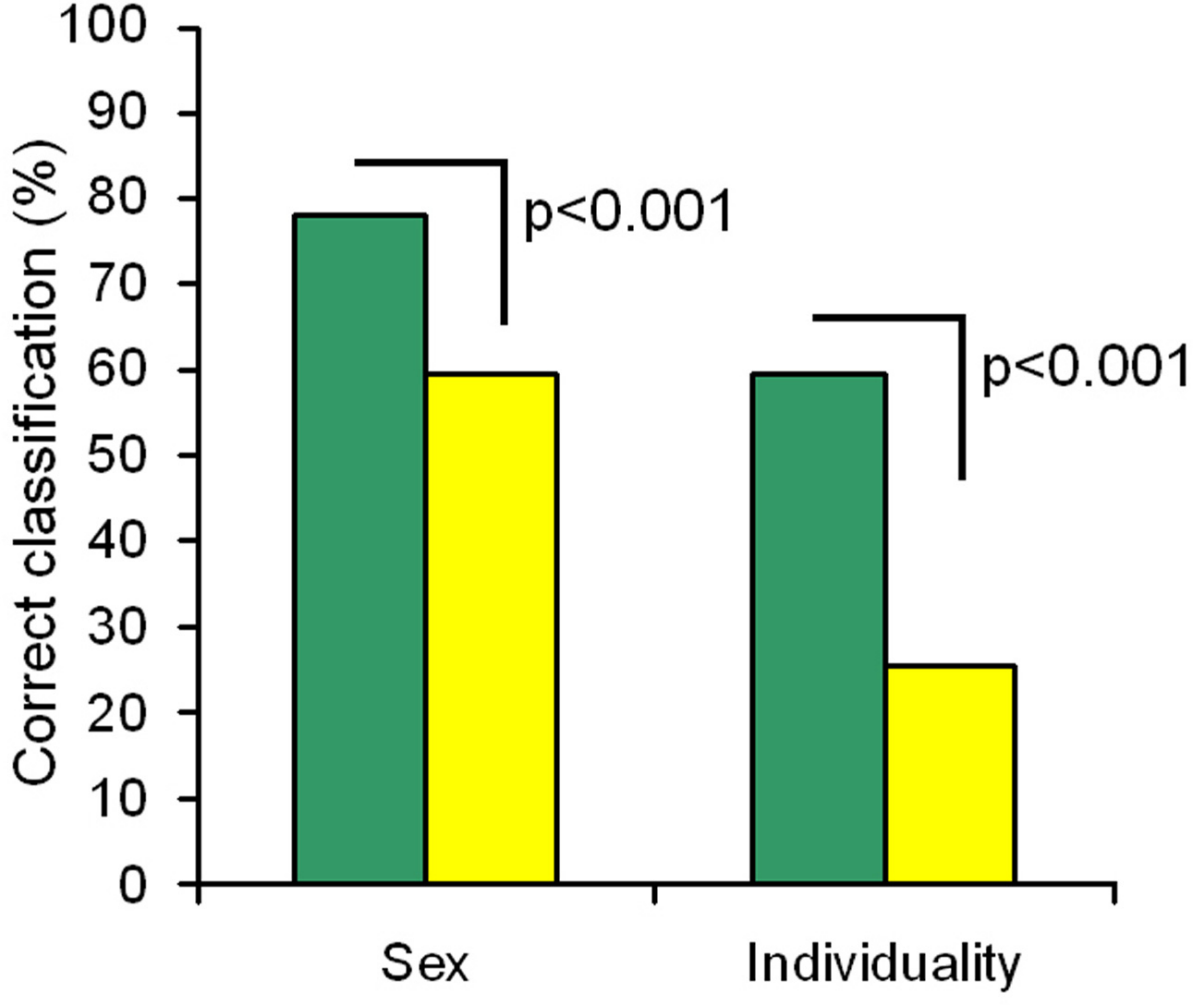
Sex and individual discrimination of the cheetah meows. Green bars indicate values of discriminant function analysis and yellow bars indicate random values, calculated with the randomization procedure. Comparisons between observed and random values with permutation tests are shown above the bars.

We conducted two DFAs (for sex and for individual identity), each DFA based on all the 9 measured variables of meows. The DFA showed the average values of correct assignment to sex of 78.1%, what was significantly higher than the random value 59.4 ± 3.3% (permutation test, 1000 permutations, *p* < 0.001) (Fig 3). In order of decreasing importance, the f0 beg, duration and q50 were mainly responsible for discrimination of sex for the meows.

At the same time, DFA showed the average values of correct assignment to individual of 59.6%, what was significantly higher than the random value 25.5 ± 3.0% (permutation test, 1000 permutations, *p* < 0.001) (Fig 3). In order of decreasing importance, the f0 max, q75 and q25 were mainly responsible for discrimination of individuals for the meows. However, the value of correct assignment varied among individuals from 20% to 73.3%, and for one of 12 individuals did not differ from the random value. Thus, meows had reliable individual-specific traits not in all individuals. Therefore, cheetah meows bear reliable cues to sex (higher fundamental frequency in females compared to males) and have a potential to encode individual identity.

### Between-year stability of meows

**Fig 4:**
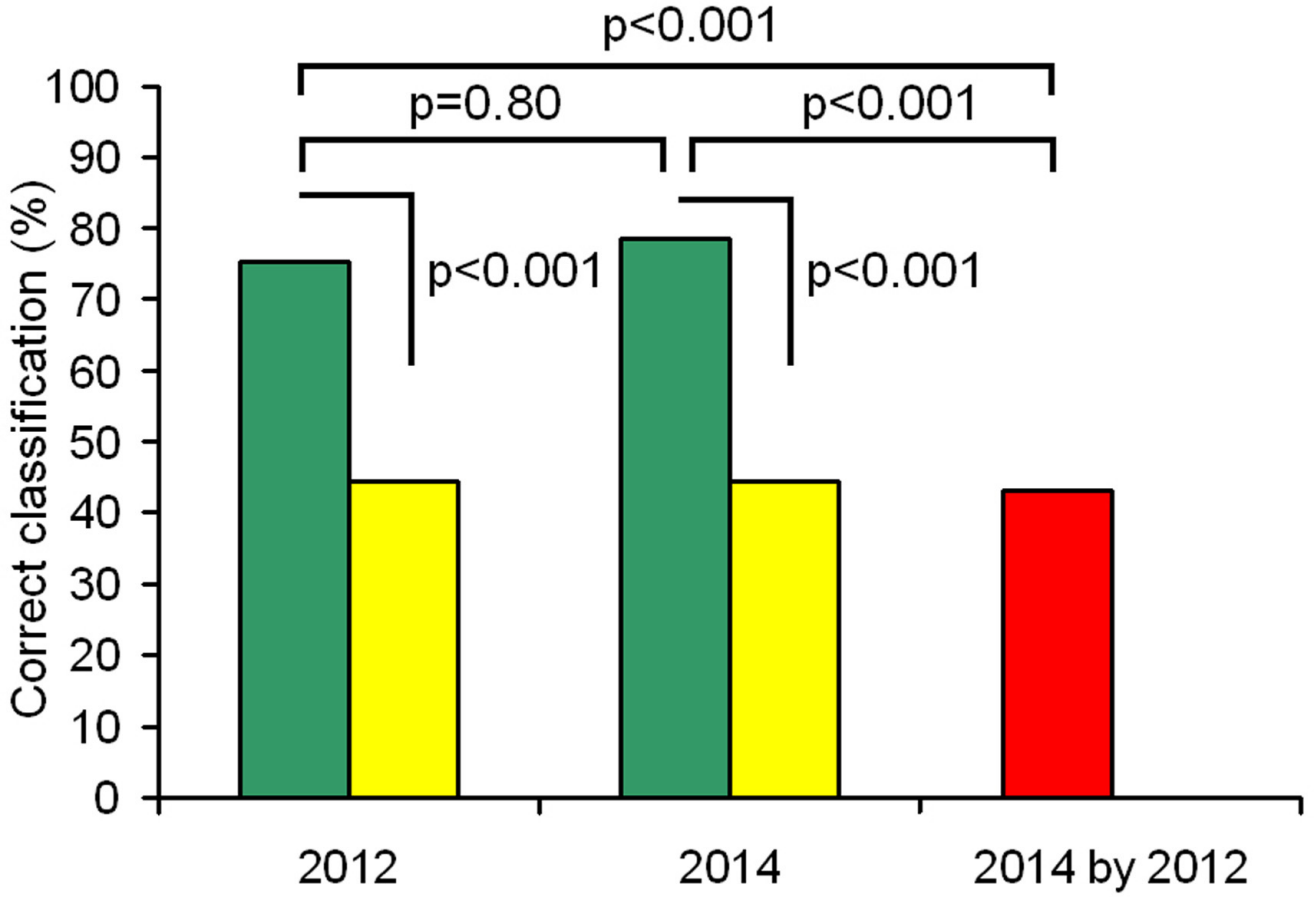
Discrimination of individual cheetahs by meows in two years (2012 and 2014). Green bars indicate values of discriminant function analysis and yellow bars indicate random values, calculated with a randomization procedure. Comparisons between observed and random values with permutation tests and comparisons between 2012 and 2014 meows with *χ*^*2*^ tests are shown by brackets above the bars. The red bar indicates the classification value of 2014 meows with discriminant functions created for meows recorded in 2012.

For 5 animals (2 males and 3 females) that provided sufficient number of meows in both 2012 and 2014, we compared the stability of vocal individuality in meows between years (Fig 4). Within years, DFA showed high values of correct classification of meows to individual (75.4% in 2012 and 78.6% in 2014) significantly exceeding the random value (44.4 ± 5.1% in 2012 and 44.8 ± 5.3% in 2014, permutation test, *p* < 0.001 in both cases), and did not differ between years (*χ*^2^_1_ = 0.06, *p* = 0.80).

However, cross-validation of meows recorded in 2014 using discriminant functions created for meows recorded in 2012, revealed a strong decrease in the correct classification of individuals (Fig 4). The average value of correct classification dropped to the level expected by chance alone (42.9%), and became significantly lower compared to call samples from either 2012 (*χ*^2^_1_ = 13.86, *p* = 0.002) or from 2014 (*χ*^2^_1_ = 17.25, *p* < 0.001). Thus, in the cheetah, individual identity of meows was unstable between years.

## Discussion

### Vocal repertoire of adult cheetahs

We found that values of acoustic variables were substantially different between call types in the cheetah. In this sense, the cheetah vocal repertoire can be considered as “discrete” vocal repertoire, similar to the more or less “discrete” vocal repertoires of other felids [37] and some ungulates, e.g. red deer [52]. This is distinctive to the more “continual” vocal repertoires, e.g., in red fox (*Vulpes vulpes*) [36] and in the wild boar (*Sus scrofa*) [61], in which a noticeable number of intermediate vocalizations occurred along to distinctive call types. Transitional forms from one call type to another occurred only in 5.8% cases, similar to [19]. No complicating features such as nonlinear phenomena or articulation effects [36,62] have been detected in the cheetah vocalizations by this or previous studies.

The values of acoustic variables were close to those found in the previous study of the cheetah vocal repertoire [19]. For instance, the average pulse rate of the chirr was 16.6 Hz in this study and 18.4 Hz in [19]; the pulse rate of the purr was 22.7 Hz in this study and 23.5 Hz in [19]; and the pulse rate of the growl was 37.4 Hz in this study and 36.4 Hz in [19], although the samples of animals, the animal housing and the staff were entirely different compared to the former study [19].

For the two phases (expiration and inspiration) of the purr vocalization, we found the differences in the pulse rate, the peak frequency, and in the lower and medium quartiles (all were significantly higher for the inspiration phase than for the expiration phase). The differences in the pulse rate (24.6 Hz during the expiration phase and 20.8 Hz during the inspiration phase) were similar with those reported by [20] (26 Hz and 21 Hz respectively), by [24] (20.9 Hz and 18.3 Hz respectively) and by [63] (21.9–23.4 Hz and 19.3–20.9 Hz respectively). The average value of pulse rate for the cheetah purr of 17.5 Hz [23] probable represents the value of pulse rate for the cheetah chirr, as in another study of this author [22] the average value of pulse rate for the cheetah chirr (termed the gurgle by [22]) was 16 Hz (ranging from 11 to 20.8 Hz).

### Contextual use of call types in the cheetah

Context-specific call types were either related to aggressive behaviour in the Offensive context (growl, howl and hiss) or to sexual behaviour in the Courting context (chirr). Consistently, earlier studies report that male cheetahs produce the chirrs when courting receptive females, whereas females use the chirrs for communication with cubs [19,64]. Towards urine samples of non-receptive (not ready to mate) females, male cheetahs remain silent [27,28]. This helps to select appropriate time for joining pairs for mating [27,28], as in zoos, males and female cheetahs are kept separately [65], otherwise they do not breed. Chirr vocalizations given towards female urine sample in the heat indicates male competence as a breeder, whereas male silence in this situation indicates its incapability to mate [27,28].

Among call types that were not context-specific (chirp, purr and meow) the meow takes an especial place in the vocal repertoire of captive cheetahs. In this study, the meow was the most often produced call type (47.6% of all calls, Table 3) presented in both sexes and in 12 of 13 individuals. In four of the 8 behavioural contexts, the meow was the most often call type, being therefore in terminology of [53] the signal-specific call type for these four contexts. Two of these contexts (Conspecific-Contact and Call-Over) are related to communication between conspecifics, therefore they might correspond to the natural usage of meows in the wild. Two remaining contexts (Release-Soliciting and Food-Anticipation) are specific for the captive conditions, as meows given in these contexts were frustration calls directed toward a keeper and appealing to human help. The regular use of meows for cheetah-human interactions may indicate manipulating the keeper behaviour by the animals. Similarly, domestic cats use meows for manipulating their owners [38,40,41]. Consistently, domestic dogs (*Canis familiaris*) exposed to insoluble tasks, appeal for help to humans, staring on them and producing specific movements [66] and frustrative whining vocalizations [67].

In contrast to the meow, the chirp was the rarest call type comprising only 0.9% of all calls, and half of them (52%, Table 3) was given in the Release-Soliciting context. In other studies, the cheetahs used chirps for calling towards cubs, mothers, potential mates or group mates [44,64]. At experimental separations of coalitions of adult males in captivity, the chirps comprised 90% of the total of 196 calls [44]. In addition, consistently to this study, both chirps and meows were used by cheetahs in contexts of food or stroll soliciting [26]. The found in this study signal-specificity of the purr for the context of friendly close-range communication with humans (Human-Contact) is consistent to reported data for the cheetah [2,19,24] and for the domestic cat [23,24].

In this study, cheetahs more often vocalized in discomfort-related contexts: the Offensive, Defensive, Release-Soliciting and Food-Anticipating contexts comprised 73.3% of calls, whereas contexts of friendly interactions between animals (Conspecific-Contact, Call-Over and Courting) or animals and humans (Human-Contact) comprised only 26.7% of all calls (Table 4). This agrees well with findings that mammals primarily use calls in contexts related to the negative emotional arousal and vocalize much more rarely when experience positive or neutral emotions [68–70].

For analysis of contextual use of call types, we used a pooled sample of calls from all cheetahs. This is the single possible approach for analysis of the contextual use of different vocalizations in either captivity or in the wild [53]. In captivity, each cheetah is situated in unique conditions that limit the potential number of possible behavioural contexts. For instance, if an animal is kept with mates, it can display towards them aggressive, friendly or sexual attitudes. Otherwise, if an animal is kept singly, any interactive contexts are impossible. Furthermore, high-ranking individuals may initiate aggressive interactions more often compared to the low-ranking individuals; some cheetahs intended to interact with people whereas others intended to avoid them. As the result, in our study different individuals participated in different sets of situations. In nature, equal time of observations for focal animals also did not help to balance call sets from different individuals by behavioural contexts [53].

### Sex and individuality in meows

We found a strong influence of sex on cheetah meow vocalizations. Sexual differences were well-expressed and mainly were determined by the values of fundamental frequency (lower in males than in females), whereas the values of all other vocal variables were indistinguishable between sexes. The found differences in fundamental frequency of about 20% (Table 5) are comparable with 15% body mass differences between males and females in captive cheetahs [8] and with 22% body mass differences between males and females in wild cheetahs [14].

However, the values of call fundamental frequency depend primarily on the length of vocal folds in the larynx [71,72], which are related in most mammals with linear body dimensions. In the cheetah, differences in linear body dimensions between males and females in the skull length, the foreleg length and the hind leg length range between 4.2 to 7.3% depending on the measure [14]. Therefore, we may expect that differences in size of the larynx between male and female cheetahs exceed the overall differences of linear body size between sexes. Earlier, sex-specific dimorphism in size of the larynx exceeding body size between males and females was reported for humans [73], Mongolian gazelles (*Procapra gutturosa*) [47], and goitred gazelles (*Gazella subgutturosa*) [48]. In humans, this dimorphism results from sexual selection for the lower-pitched male voices as a component of apparent body size exaggeration for attracting females and competing with males [74,75]. Nevertheless, in the cheetah, voice pitch differences reliably reflect the differences in body size between sexes, as in most mammalian and bird species with sex dimorphism of body size [76,77], but see [51,52].

In cheetah meows, sex differences were higher whereas individual differences were comparable to other mammals, in which DFAs to identity and sex were applied to the same samples of calls and animals. Sex discrimination higher than random was reported for alarm calls of adult yellow-bellied marmots (*Marmota flaviventris*) [78], for contact calls of young goitred gazelles [79] and for alarm calls of giant otters (*Pteronura brasiliensis*) [80]. At the same time, in alarm calls of speckled ground squirrels (*Spermophilus suslicus*) [78], yellow ground squirrels (*S*. *fulvous*) [78] and chinchillas (*Chinchilla lanigera*) [81] and in barks of two borzoi breeds of the domestic dog [35], discrimination to sex was found on the level expected by chance alone, whereas individual differences were comparable to those in cheetah meows in our study.

Although vocal individuality was well-expressed in cheetah meows, these individualistic features were unstable over years. Indeed, all studied species of mammals display poor stability of individual vocal traits with time: speckled and yellow ground squirrels [56,60,82], domestic dogs [35], red deer [58,83], fallow deer (*Dama dama*) [57] and common marmosets (*Callithrix jacchus*) [84]. In contrast, stable individual and pair duet vocal signatures were found for the periods up to five years in some birds: red-breasted geese (*Branta ruficollis*) [85], red-crowned cranes (*Grus japonensis*) [86] and crested auklets (*Aethia cristatella*) [87]. It seems that bird calls retain better the individualistic traits compared to calls of mammals, however further study with more species and call types is necessary to confirm this.

### Vocal repertoires of adult and cub cheetahs

The vocal repertoire of the cheetah is primarily stated at birth, as all seven call types (chirr, growl, meow, chirp, howl, purr and hiss) described in adults in this study and in [19] were previously described in 15 cheetah cubs aged from 2 days to 3 months [18]. However, whereas the entire set of call types is already presented in cubs, the acoustic variables changed strongly with age [18,19]. In vocal repertoire of 1.5–3 months-old cubs [18], the average duration of the chirr was 0.42 s (n = 19 chirrs), that is much shorter than 0.74 s in adults in this study (Table 1); the average duration of the growl was 0.93 s (n = 24 growls), that is much shorter than 2.34 s in adults in this study (Table 1), and the average duration of the howl was 0.70 s (n = 6), that is much shorter than 2.11 s in adults in this study (Table 1). At the same time, the average duration of cub meow was 0.56 s (n = 38 meows), that is substantially longer than 0.32 s in adults in this study (Table 1). Consistently, the average duration of cub chirp was 0.32 s (n = 139 calls), that is much longer than 0.11 s in adults in this study (Table 1).

The fundamental frequency variables also differed between the 1.5–3 months-old cheetah cubs [18] and adults in this study. The average maximum fundamental frequency of cub meows was 3.89 kHz (n = 38 meows) that is much higher than 0.94 kHz in adults in this study (Table 1). The average maximum fundamental frequency of cub chirps was 5.85 kHz (n = 142 chirps) that is much higher than 1.81 kHz in adults in this study (Table 1). The average maximum fundamental frequency of cub howls was 2.58 kHz (n = 6) that is much higher than 0.31 kHz in adults in this study (Table 1).

For the cheetah, the substantially higher values of maximum fundamental frequency in cubs than in adults indicate the descending ontogeny of fundamental frequency with age that is usual for mammals [76,88], some birds [89–91] and reptiles [92]. The distinctive ontogenetic pathways with same-frequency or even lower-frequency calls in the young than in adults were reported for the Siberian red deer (*Cervus elaphus sibiricus*) [52], four species of ground squirrels [88,93–95] and two species of shrews [96–98].

## Conclusion

All studies of cheetah vocalizations including the present study have been conducted in captivity. Captive conditions hardly affect the acoustic variables given that cheetah vocal repertoire is stated at birth. However, the captive conditions might affect somehow the contextual use of vocalization by these animals. Captive conditions include some behavioural contexts that do not occur in nature (e.g, the contexts involving animal-human communication). At the same time, some natural contexts may lack in captivity. Further research of vocal behaviour of free-ranging cheetahs should reveal the natural use of each call type in different ages and sexes of cheetahs.

## Supporting Information

S1 Audio Calls of adult cheetahs. Purr, hiss, growl, chirr, meow, chirp, howl.
(WAV)

S1 Table Acoustic measurements of cheetah seven call types for describing the acoustics of call types. (XLS)

S2 Table Acoustic measurements of cheetah meows for estimating the effects of sex and individuality on the acoustic variables of meows and for estimating the stability of vocal individuality in meows. (XLS)
